# Shift work and risk of incident gastroesophageal reflux disease: the association and mediation

**DOI:** 10.3389/fpubh.2023.1192517

**Published:** 2023-08-24

**Authors:** Qian Li, Fu-Rong Li, Shihan Zhen, Jian Liao, Keye Wu, Xia Li, Bincai Wei, Zhiyi Xiao, Qingyao Wu, Xian-Bo Wu, Fengchao Liang

**Affiliations:** ^1^School of Public Health and Emergency Management, Southern University of Science and Technology, Shenzhen, China; ^2^Department of Epidemiology, School of Public Health, Southern Medical University, Guangzhou, Guangdong, China

**Keywords:** shift work, gastroesophageal reflux disease, cohort study, mediation, public health, risk factors

## Abstract

**Introduction:**

Shift work has become an increasingly common work mode globally. This study aimed to investigate the association between shift work and the risk of incident gastroesophageal reflux disease (GORD), an upward gastrointestinal disorder disease worldwide, and to explore the mediating factors.

**Method:**

A total of 262,722 participants from the UK Biobank free of GORD and related gastrointestinal diseases were included to investigate the association and potential mediators between shift work and incident GORD. Multivariate-adjusted Cox models were used to evaluate the association between shift work status and GORD incidence.

**Results:**

Compared to non-shift workers, shift workers had a 1.10-fold greater risk of incident GORD [95% confidence intervals (CIs): 1.03, 1.18], after adjusting for a range of potential confounders. However, the excess risk of GORD attenuated to the null after further adjusting for selected mediators. Specifically, the association was mediated by sleep patterns (25.7%), healthy behaviors (16.8%), depressive symptoms (20.2%), chronic conditions (13.3%), and biological factors (17.6%). After adjustment for all the mediators together, the association was attenuated by 71.5%.

**Discussion:**

Our findings indicated that long-term shift workers may have a higher risk of incident GORD, yet the excess risk may be explained by poor sleep quality, unhealthy behaviors, depressive symptoms, etc. This has positive implications for protecting the health of shift workers.

## 1. Introduction

Shift work, which is defined as rotating work patterns that include evening, overnight or weekend employment (1), is one of the elements influencing different lifestyle behaviors and illness risks associated with diverse employment (2–4). Due to the strengthening of 24-h service demand and globalization, shift work is very common in various occupational areas, with approximately 21% of the labor force in Europe employed in this pattern (1). It is believed that this unusual employment status disrupts internal circadian rhythms (5) and may lead to a higher risk of chronic diseases, such as psychiatric disorders (6), cardiovascular disease (7) and gastrointestinal complaints (8).

Gastroesophageal reflux disease (GORD) is a widespread gastrointestinal disorder with an estimated prevalence of 14% globally, and over 20% in developed countries in 2020 (9). GORD causes esophageal symptoms and complications, and even extraesophageal symptoms such as hoarseness, wheezing, cough, and asthma by gastric juice reflux (10).

In addition to the physiological effects, it is found that GORD patients may experience an increase in the incidence of depression and anxiety (11), accompanied by a decrease in work efficiency (12). However, epidemiologic evidence on the association between shift work and incident GORD risk is limited. Only a few cross-sectional studies reported shift work to be associated with prevalent GORD (13–15). A recent meta-analysis that included four cross-sectional studies of shift work and GORD also reported a pooled significant positive association (OR = 1.53; 95% CI: 1.33, 1.77) (16). These findings, however, were based on cross-sectional nature, failing to assess the causal association. In addition, previous studies have suggested that sleep disturbance (17), obesity (18), unhealthy diet patterns (19), and psychological disorders (20) are risk factors for the development of GORD. Whereas, at the same time shift work has been shown in previous studies to contribute to above risk factors of GORD incidence (21–23). The relationship between shift work and GORD may be mediated due to these factors, but whether and to what extent various factors may mediate this association remains unclear. Indeed, mediators can be early warning indicators of disease progression and help to understand potential pathways, which have not been reported between shift work and incident GORD yet.

Therefore, our study aimed to investigate the association between shift work and the risk of GORD incidence by using the UK Biobank study, a large-scale cohort study in the United Kingdom. We also examined the effect of mediators such as sleep patterns, health behaviors, depressive symptoms, and biological factors on the risk of incident GORD.

## 2. Methods

### 2.1. Study population

The UK Biobank is a population-based longitudinal study of ~ 0.5 million participants aged 40–69 y, who were enrolled between 2006 and 2010 from 22 health assessment centers across the U.K.. Details of the UK Biobank have been extensively described elsewhere (24). During baseline assessment, a wide range of health-related information was collected from touchscreen questionnaires, physical measurements, and biological samples. The UK Biobank study was approved by the North West Multi-Center Research Ethics Committee, and all participants provided written informed consent. At the time of our analysis, hospital inpatient records were available until 30th November 2020, and mortality data were updated to 26th April 2020 for England and Wales and 20th April 2020 for Scotland; consequently, these dates were utilized as the end of follow-up.

**Figure 1:**
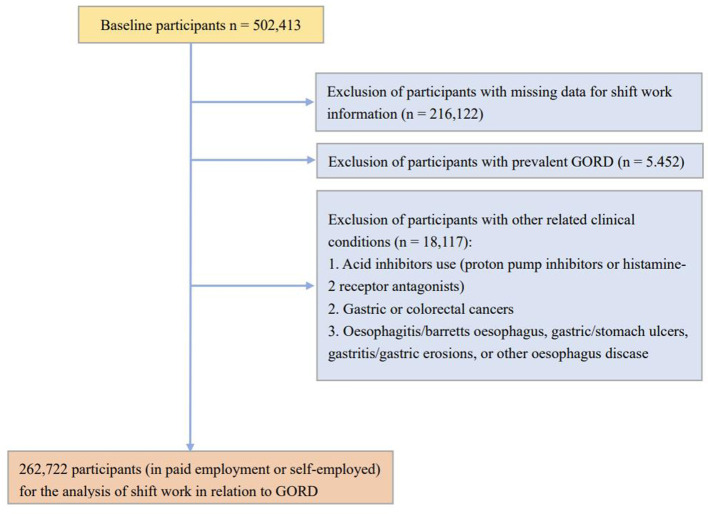
The selection criteria of study population.

In the present study, we initially included 287,086 participants who were in paid employment or self-employed at baseline assessment. We further excluded those with missing data for shift work information (*n* = 795) or with prior GORD history (*n* = 5,452). To exclude potential GORD history and avoid reverse causality, we also excluded those with GORD-related clinical conditions (*n* = 18,117), including acid inhibitors use (proton pump inhibitors or histamine-2 receptor antagonists), gastric or colorectal cancers, oesophagitis/Barrett's esophagus, gastric/stomach ulcers, gastritis/gastric erosions, or other esophagus disease. A total of 262,722 participants were included for the analysis (Figure 1).

### 2.2. Shift work assessment

Participants who were in paid employment or self-employed were inquired whether their current job involves shift work and night-shift work, walking or standing, and heavy manual or physical work, with answer choices including “never/rarely,” “sometimes,” and “usually or always”. Shift work referred to a work schedule that fell outside of the typical daytime working hours of 9 a.m.−5 p.m., which may include working afternoons, evenings or nights, or rotating through these kinds of shifts. In our analysis, the exposure to shift work was defined as a job requiring participants to sometimes, usually or always work shifts. As such, no shift work was defined as jobs that rarely/never require shift work. Additionally, shift work was also classified according to shift work frequency (always vs. sometimes/usually) and whether shifts were night shifts (shifts sometimes/usually/always at night) or evening/weekend shifts (never/rarely at night). The duration of shift work was defined as the duration of the current employment for shift workers.

### 2.3. GORD assessment

GORD was defined by using self-reported diagnoses, diagnostic (ICD-10)/procedural codes (OPCS4) linked to hospital inpatient records, and death registers (25). Self-reported diagnoses were used to ascertain prevalent cases only. The following ICD-10 codes were utilized: K21.9 (GORD without esophagitis), K21.0 (GORD with esophagitis). Additionally, OPCS4 operation code of G24 (anti-reflux operations) and G25 (revision of anti-reflux operations) were also used. Person-time in Cox models was calculated from the date of baseline assessment to the date of diagnosis of the event, death, the end of follow-up, or lost to follow-up, whichever came first.

### 2.4. Assessment of covariates/potential mediators

At baseline assessment, participants completed touch-screen questionnaires and then were interviewed by a trained investigator face-to-face. We reviewed the literature about the factors associated with both shift work and GORD, and decided to select a set of individual-level variables as possible mediators that may result in over-adjustment in the present study, because they were possible mediators and may be the pathway between shift work and incident GORD. The variables were generally grouped into six categories: demographic and socioeconomic factors, sleep patterns, health behaviors, depressive symptoms, chronic conditions, and biological factors. Details regarding the definitions of covariates/potential mediators are presented in the Supplementary material 1.

#### 2.4.1. Demographic and socioeconomic factors

This domain included basic demographics (age, sex), ethnicity (White, Black or Black British, Asian or Asian British, Mixed, Chinese, other ethnic groups) and socioeconomic factors (education levels and Townsend deprivation index). Education levels were grouped into elementary school and below, secondary education and university degree. The Townsend deprivation index is a composite score reflecting socioeconomic deprivation and household income (26).

#### 2.4.2. Sleep patterns

Information on sleep patterns was collected by touchscreen questionnaires. The number of sleep disturbances included five circadian and sleep characteristics: late chronotype, inadequate sleep duration ( ≤ 7 h/day or ≥8 h/day), usually insomnia, snoring, and frequent daytime sleepiness. Healthy sleep patterns were defined as adequate sleep duration and free of sleep disturbance.

#### 2.4.3. Heath behaviors

Health behavioral factors, including smoking status, alcohol consumption, diet habit, physical activity, and daily sedentary time were self-reported. Physical activity was defined as regular based on various physical activity domains. Alcohol consumption (g/d) was estimated by 18 questions regarding intakes of beer, wine (red and white) and spirits, the standard to sum up alcohol consumption is presented in the Supplementary material 1. Smoking status was grouped into three categories: never, current and previous smoke. Sedentary time (hours) was a composite variable involving time spending on driving, using a computer, and watching television per day. Habitual diet was assessed through a food frequency questionnaire. The present study used a healthy diet score that comprised a range of food intake. Health behaviors used for mediation analysis included current smoking, regular exercise and sedentary time. Healthy behaviors were defined as not current smoking, regular exercise and sedentary time less than the median values.

#### 2.4.4. Depressive symptoms

Depressive symptoms were assessed using the frequency of four indicators in the previous 2 weeks of interview (27): (1) depressed mood, (2) disinterest or absence of enthusiasm, (3) tenseness or restlessness, and (4) tiredness or lethargy. No depressive symptoms were defined as free of those symptoms.

#### 2.4.5. Chronic conditions

Long-term illness was captured by a touchscreen question “Do you have any long-standing illness, disability or infirmity?”. The number of medications (treatments) was also reported. In the present study, polypharmacy was defined as ≥ 5 medications (treatments). No chronic conditions were defined as free of chronic conditions such as diabetes or hypertension.

#### 2.4.6. Biological factors

Biological factors included body mass index (BMI), waist to hip ratio (WHR), grip strength, glycated hemoglobin (HbA1c), C-reactive protein (CRP), gamma-glutamyltransferase (GGT) (U/L) and estimated glomerular filtration rate (eGFR). BMI was used to reflect general obesity while WHR was a marker of central obesity. Grip strength was a proxy for muscle weakness. HbA1c, CRP, CGT and eGFR were used as markers of glycemic control, systemic inflammation, liver function and kidney function, respectively. Measurements of biomarkers were validated and details were provided elsewhere (28). Healthy biological factors were defined as adequate indicators of selected biological factors.

#### 2.4.7. Statistical analysis

Baseline characteristics of the study participants were described as mean ± standard deviation for continuous variables and number (percentage) for categorical variables. Cox proportional hazard models were used to estimate the hazard ratios (HRs) and 95% confidence intervals (CIs) for incident GORD associated with shift work status. The proportional hazards assumption was tested by creating a time-dependent variable and no violation was observed. In model 1, age, sex, ethnicity, Townsend deprivation index education level, hours of work per week, duration of current job, walking/standing at work and heavy manual/physical were adjusted. In model 2, BMI, healthy diet score and alcohol consumption were further adjusted.

To examine the extent to which the above-mentioned sleep patterns, health behaviors, depressive symptoms, chronic conditions and biological factors explained the associations of shift work and GORD incidence, the percentage of excess risk mediated (PERM) was estimated by the following equation (29, 30): *PERM* = [*HR*(*model* 2)−*HR*(*model* 2 *plus variable group*)]/ [*HR*(*model* 2)−1]. This method could provide the magnitude of the mediation effect of a group of various variables as a whole in a Cox model. Additional mediation analysis was also conducted to evaluate the proportional association of each single factor within the association between shift work status and incident GORD with the mediation package in R software (31). Complete case analysis was conducted if there were missing data.

We conducted subgroup analyses stratified by basic sociodemographic factors and work-related factors, including age, sex, education attainment (with or without a university degree), Townsend deprivation index (High: <-2.08, middle: ≥-2.08 to <1.40 or low: ≥1.40), hours of work per week (<37 or ≥37 h), job involves walking or standing (never or rarely, sometimes or more), and heavy manual labor (never or rarely, or sometimes or more). To avoid over-adjustment, subgroup analyses was based on model 2.

Analyses were conducted using Stata version 14.0 (College Station, Texas) and R version 3.4.2 (R foundation for Statistical Computing).

## 3. Result

The number of participants included were 262,722, of which 22,622 always worked in night shift and 21,766 always worked in the evening or weekend shift. Generally, night shift workers were younger, worked for longer hours per week and were less likely to be white and female than non-shift workers. Compared to the no shift work group, evening/weekend shift and night shift employees performed more manual or physical labor work (60.2 and 71.2%, respectively) and tended to suffer from depressive symptoms such as depression and tension; their BMI was also higher, and they had lower healthy diet score. Additionally, shift workers had shorter sleep duration and more sleep disturbances, compared with non-shift workers (Table 1).

**Table 1:** Baseline characteristics of the study participants in the UK Biobank for the analysis of association between shift work and the risk of GORD.

	**No shift work (%)**	**Evening/weekend shift (%)**	**Night shift (%)**
**Demographic and socioeconomic factors**			
Age, y	52.7 ± 7.1	52.3 ± 7.0	51.1 ± 6.8
College or university degree	90,573 (42.0)	5,451 (25.5)	4,645 (20.9)
Townsend deprivation index	−1.5 ± 2.9	−0.6 ± 3.2	−0.5 ± 3.3
Women	117,264 (53.7)	11,433 (52.5)	8,573 (37.9)
White	207,330 (95.0)	19,719 (90.6)	19,711 (87.1)
**Work related factors**			
Heavy manual/physical labor at work	61,050 (28.0)	13,094 (60.2)	16,098 (71.2)
Number of work hours a week	34.8 ± 12.5	35.8 ± 12.3	40.3 ± 13.1
Walking or standing at work	130,742 (59.9)	18,257 (83.9)	19,594 (86.7)
Years working in current job	13.5 ± 10.5	12.9 ± 10.2	14.5 ± 10.7
**Sleep patterns**			
Sleep duration 7–8 h/d	156,714 (71.9)	14,191 (65.5)	13,341 (59.4)
>2 sleep disturbances	66,942 (30.7)	7,276 (33.4)	8,585 (38.0)
**Heath behaviors**			
Alcohol consumption, g/d	15.1 ± 17.9	15.1 ± 20.1	16.9 ± 22.0
Current smokers	21,178 (9.7)	3,033 (13.9)	3,738 (16.5)
Healthy diet score	2.8 ± 1.4	2.7 ± 1.4	2.5 ± 1.4
Regular exercise	162,682 (75.3)	16,922 (79.4)	17,996 (81.4)
Sedentary time, h/day	4.6 ± 2.4	5.0 ± 2.8	5.5 ± 3.1
**Depressive symptoms**			
Ever had depressed mood	48,170 (22.9)	5,558 (27.0)	5,349 (25.1)
Ever felt tense in the past two weeks	55,538 (26.3)	6,203 (30.0)	5,995 (28.0)
Ever had little interest in doing things	42,468 (20.0)	5,349 (25.8)	5,840 (27.2)
Ever felt tired	115,447 (54.2)	12,293 (58.7)	12,593 (57.8)
**Chronic condition**			
Long-standing illness	48,583 (22.3)	5,552 (25.5)	5,462 (24.1)
Taking ≥5 medications	19,420 (8.9)	2,172 (10.0)	2,033 (9.0)
**Biological factors**			
BMI, kg/m^2^	27.0 ± 4.6	27.6 ± 5.0	28.2 ± 4.9
C-reactive protein, mg/L	2.4 ± 3.8	2.6 ± 3.7	2.7 ± 4.0
eGFR, ml/min/1.73 m^2^	94.7 ± 12.6	95.7 ± 12.8	96.7 ± 12.9
Gamma-glutamyltransferase, U/L	34.7 ± 36.0	35.7 ± 35.5	35.5 ± 37.4
Handgrip strength, kg	32.4 ± 10.9	32.0 ± 11.0	35.3 ± 11.3
HbA1c, mmol/L	35.1 ± 5.9	35.7 ± 6.6	35.9 ± 7.0
Waist to hip ratio	0.9 ± 0.1	0.9 ± 0.1	0.9 ± 0.1

BMI, body mass index; eGFR, estimated glomerular filtration rate; GORD, gastroesophageal reflux disease; HbA1c, glycated hemoglobin.

Table 2 shows the association between shift work and incident GORD risk. After adjusting for demographic and socioeconomic factors, work-related factors, BMI, healthy diet score and alcohol consumption, the HR of incident GORD was 1.10 (95% CI: 1.03, 1.18) for shift workers, in comparison to non-shift workers. Based on model 2, those who reported sometimes or always shift work had elevated risk of incident GORD (Table 3), with corresponding adjusted HR (95% CI) of 1.09 (1.01, 1.18) and 1.11 (1.02, 1.21), respectively; also, night shift workers had a higher risk of incident GORD (HR = 1.15, 95% CI: 1.06, 1.25). After review of relevant studies, we selected five groups of potential mediators, including sleep patterns, health behaviors, depressive symptoms, chronic conditions and biological factors. Notably, after adjustment for all five groups of protective mediators, the associations of shift work, frequency of shift, and type of shift with the risk of GORD attenuated to the null (Tables 2, 3); while the links between years of shift work became worth noticing (Supplementary Table 1). We also present the subgroup analyses for the association of shift work and GORD risk. The increased risk of incident GORD associated with shift work was observed among those who reported having never or rarely heavy manual labor only (*P* for interaction = 0.002) (Supplementary Table 2).

**Table 2:** Association and proportions of the shift work and GORD excess risk mediated by mediator groups.

	**HR (95% CI)**	***P-*value**	**PERM**
Model 1	1.13 (1.05, 1.21)	0.001	-
Model 2	1.10 (1.03, 1.18)	0.007	-
+ Sleep patterns	1.07 (1.00, 1.15)	0.043	25.7%
+ Heath behaviors	1.08 (1.01, 1.16)	0.027	16.8%
+ Depressive symptoms	1.08 (1.00, 1.16)	0.042	20.2%
+ Chronic condition	1.09 (1.01, 1.17)	0.018	13.3%
+ Biological factors	1.08 (1.01, 1.17)	0.034	17.6%
All	1.03 (0.95, 1.12)	0.493	71.5%

Model 1 was adjusted for age, sex, ethnicity, Townsend deprivation index and education, hours of work per week, duration of current job, walking/standing at work and heavy manual/physical work.

Mode 2 was further adjusted for BMI, healthy diet score, and alcohol consumption.

Sleep patterns: chronotype and number of sleep disturbance.

Heath behaviors: current smoking, regular exercise, sedentary time.

Depressive symptoms: depressed mood, disinterest or absence of enthusiasm, tenseness or restlessness and tiredness or lethargy in the previous 2 weeks.

Chronic condition: long-standing illness and number of medications use.

Biological factors: C-reactive protein, eGFR, grip strength, gamma-glutamyltransferase, HbA1c, waist to hip ratio.

PERM, percentage of excess risk mediated, calculated with healthy sleep patterns, healthy behaviors, no depressive symptoms, no chronic conditions, healthy biological factors and all together (defined in Method).

**Table 3:** Associations of frequency and type of shift work with the risk of incident GORD.

**Frequency of shift**	**HR (95% CI)**	***P-*value**
**Model 1**		
Sometimes	1.12 (1.03, 1.21)	0.006
Always	1.14 (1.05, 1.24)	0.002
**Model 2**		
Sometimes	1.09 (1.01, 1.18)	0.027
Always	1.11 (1.02, 1.21)	0.012
**Model 2 plus all potential mediators**		
Sometimes	1.03 (0.94, 1.13)	0.476
Always	1.02 (0.93, 1.13)	0.649
**Type of shift**	**HR (95% CI)**	***P-*** **value**
**Model 1**		
Evening/weekend shifts	1.08 (1.00, 1.17)	0.053
Night shifts	1.19 (1.09, 1.29)	< 0.001
**Model 2**		
Evening/weekend shifts	1.06 (0.98, 1.15)	0.139
Night shifts	1.15 (1.06, 1.25)	0.001
**Model 2 plus all potential mediators**		
Evening/weekend shifts	1.00 (0.91, 1.09)	0.920
Night shifts	1.07 (0.97, 1.18)	0.151

Model 1 was adjusted for age, sex, ethnicity, Townsend deprivation index and education.

Model 2 was further adjusted for hours of work per week, duration of current job, walking/standing at work and heavy manual/physical work.

CI, confidence interval; HR, hazard ratio.

Based on the model with adjustment for demographic and socioeconomic factors and work-related factors, we further explored the mediating effects of several groups of potential mediators, namely, healthy sleep patterns, healthy behaviors, no depressive symptoms, no chronic conditions, healthy biological factors and all factors as a whole, with the association between shift work and incident GORD attenuated by 25.7, 16.8, 20.2, 13.3, 17.6, and 71.5%, respectively (Table 2). Table 4 shows the mediation analysis of the association between shift work and incident GORD. Among each single potential mediator, sedentary behavior is the largest mediator (15.09%), followed by sleep duration (13.01%), ever felt depressed (11.52%), and ever felt tense (9.89%), etc. Details of the mediation analysis are also provided (Supplementary Tables 3, 4).

**Table 4:** Summary of mediation analysis between shift work and the risk of GORD.

**Variable**	**Shift work**	**GORD**	**% mediated (95% CI)**
**Sleep patterns**			
Sleep duration	−	−	13.01 (6.72, 24.0)
Sleep disturbance	−	−	9.62 (6.05, 40.00)
**Heath behaviors**			
Current smoking	+	+	4.48 (1.97, 21.22)
Regular exercise	−	−	1.51 (0.03, 5.31)
Sedentary time	+	+	15.09 (9.47, 63.23)
**Depressive symptoms**			
Ever felt depressed	+	+	11.52 (7.68, 39.80)
Ever felt tense	+	+	9.89 (5.99, 56.77)
Ever had little interest in doing things	+	+	11.07 (6.83, 76.22)
Ever felt tired	+	+	9.37 (5.36, 56.81)
**Chronic condition**			
Long-standing illness	+	+	7.64 (4.25, 36.31)
Taking ≥5 medications	+	+	4.48 (1.97, 21.16)
**Biological factors**			
C-reactive protein	+	+	0.32 (0.020, 1.11)
eGFR	+	0	−0.05 (−0.50, 0.01)
Gamma glutamyltransferase	+	+	0.68 (0.0082, 4.27)
Grip strength	−	−	5.15 (3.06, 21.32)
HbA1c	+	−	−1.89 (−12.79, −1.11)
Waist to hip ratio	+	+	3.52 (1.88, 15.01)

+, Positive association; −, negative association; 0, no association; eGFR, estimated glomerular filtration rate; GORD, gastroesophageal reflux disease; HbA1c, glycated hemoglobin.

## 4. Discussion

In this longitudinal cohort study of 262,722 participants in the UK, we found that shift workers may have a higher risk of incident GORD than day workers, particularly among those with long-term shift work. A wide range of factors, including sleep patterns, health behaviors, depressed symptoms, chronic conditions as well as biological indicators may largely mediate the associations.

Our findings regarding shift work leads the risk of incident GORD were generally consistent with those of previous cross-sectional studies (13–15). Xue et al. (15) conducted a cross-sectional investigation on rotating night shift employment and GORD in 2,027 workers, and found that rotating night shift work was associated with prevalent GORD, with an odds ratio (OR) of 3.66 (95% CI: 2.52, 5.40). Using data from 15,283 outpatients, Li et al. (14) also reported that night shift workers had a 1.38-fold higher risk of having GORD, compared with non-shift workers, and the OR was 1.38 (95% CI: 1.11, 1.71). These previous studies, however, did not adjust for potential confounders, such as BMI, alcohol consumption and diet habits (32–34), which may result in risk bias. Moreover, since cross-sectional surveys provided weak evidence on causal effect, our longitudinal study with a much larger sample size would provide a relatively high-quality health effect evidence in this field.

Our study also reported the potential mediators between shift work and incident GORD, which may provide empirical evidence to further investigate the mechanisms. Indeed, the present study assessed five mediator groups, that is, sleep patterns, health behaviors, depressive symptoms, chronic conditions, and biological factors, and found that approximately 71.5% of the excess risk of incident GORD associated with shift work was attributable to these factors all together (Table 2). Thus, the risk of incident GORD can be avoided by modifying these mediators for shift workers, and by modifying other mediators for clinical patients with existing mediators, for example, patients with insomnia could do regular exercise and avoid sedentary to attenuate the risk of GORD incidence. In addition, shift work can be considered as a risk factor when treating patients with GORD, and moderating these mediators can be used as intervention and treatment for shift workers with GORD. Of these potential mediators, sleep patterns mediated about 25.7% of the association, and further analysis found that sleep duration and sleep disturbance may contribute to about 13.01 and 9.62% of the mediation effects, respectively (Table 4). Shift workers always work at night and previous studies have demonstrated that gastric acid output reaches the highest at approximately 10 P.M. and lowest at ~7 A.M. (35). Studies have shown that long-term disruptions in circadian rhythms frequently result in a variety of gastrointestinal (GI) dysfunctions, including microbiota dysbiosis and GORD (36). Alterations in circadian rhythms, typically altered sleep or eating times, can significantly affect optimal GI function (36, 37). Furthermore, insufficient sleep time and sleep disturbance-lead insufficient sleep duration will enhance the stimulation of gastric acid in the esophagus, hence raising the potential risk of incident GORD (38). Previous research has also shown that the melatonin levels of late chronotypes are higher than those of early chronotypes (39, 40). Endogenous melatonin can also protect esophageal mucosal to minimize reflux incidence (41, 42). Consequently, the human body has a unique defense system against this circadian disturbance, but it cannot counteract the elevated risk of incident GORD (HRs: 1.09; 95% CI: 1.05, 1.21). As such, adequate sleep duration and good sleep quality may also help attenuate the risk. Insufficient sleep time and sleep disturbance-lead insufficient sleep duration could enhance the stimulation of gastric acid in the esophagus, hence raising the potential risk of incident GORD (38). Our findings also indicated that health behaviors, which had a moderating effect of 16.8%, may be a significant mediator. Of these, sedentary time contributed a mediating effect of 15.09%. Previous cohort studies on the association between lifestyle and GORD found lack of exercise to be an independent risk factor for GORD (43), whereas regular exercise has a protective effect against GORD (44).

The present study also found that psychological factors, namely, depressed symptoms, may also play an important role in the pathway between shift work and GORD incidence, and have negative effect on the risk. Mechanistically, the link between mental health and GORD incidence may cause by the influence of psychological factors on sleep quality (45). On the other hand, suboptimal psychological health may be linked to lower esophageal sphincter pressure, increased gastric acid secretion, and reduced acid clearance in the esophagus (45, 46), leading to an increased risk of GORD incidence. Furthermore, fatigue has also been demonstrated to mediate the excess risk in this study. Wang et al. (47) showed that fatigue and stress may be linked to the episodes of GORD by using a population-based study of 2,789 participants. Therefore, our study may suggest that it is necessary to advocate regular psychological intervention and work stress relief for shift workers.

This is, to our knowledge, the first large cohort study to report the association between shift work and the risk of incident GORD in the UK and to assess a wide range of potential mediators that may be involved in the development of GORD among shift workers. Our study also has some limitations. First, we could not adjust for the interplay of mediators such as psychological factors and sleep patterns, thus the impact of psychological disorders and sleep problems may be exaggerated because of their mutual influences. Second, participants in the UK Biobank cohort are basically white, and the age is concentrated between 40 and 69 years old, so the results may not be generalizable to other races or ages. Furthermore, it is noteworthy that our results may be influenced by the 'health worker effect (HWE),' meaning that the unhealthy population may have a lower chance of employment, leading to potential selection bias. Previous studies have indicated that the HWE can bias relative risk estimates toward the null value (48, 49). Consequently, the null findings in our study may be attributed to the healthy selection bias, and replication of our results in other populations is vital to confirm the associations. Finally, GORD was confirmed by self-reported diagnoses and hospital inpatient records, which might have led to outcome misclassification and underestimation of the true associations.

## 5. Conclusion

Although long-term shift workers may have a higher risk of incident GORD, most of the excess risk may be explained by poor sleep quality, unhealthy behaviors, depressive symptoms, etc. Our findings indicated that lifestyle counseling, insomnia treatment and psychological therapies for shift workers might largely adjust the risk of developing GORD. This has positive implications for protecting the health of shift workers, and guides employers to provide medical coverage and livelihood benefits for shift workers to avoid developing GORD.

## Patient and public involvement

Patients were involved in this research through data collection from the UK Biobank.

## Data availability statement

The original contributions presented in the study are included in the article/Supplementary material, further inquiries can be directed to the corresponding authors.

## Ethics statement

The studies involving humans were approved by the North West Multi-Centre Research Ethics Committee. The studies were conducted in accordance with the local legislation and institutional requirements. Written informed consent for participation was not required from the participants or the participants' legal guardians/next of kin in accordance with the national legislation and institutional requirements.

## Author contributions

QL contributed to the statistical analyses and had primary responsibility for writing the manuscript. F-RL contributed to the statistical analyses and had responsibility for method writing. FL and F-RL designed and directed the study and also provided overall supervision. F-RL and KW contributed to the data cleaning and interpretation of the data. SZ, JL, XL, BW, ZX, QW, and X-BW participated in data analysis and critical revision of the manuscript. All authors read and approved the final paper.
