# Supplementary material for: Shift work and risk of incident gastroesophageal reflux disease: the association and mediation
Source: Front Public Health. 2023 Aug 24;11:1192517. doi: 10.3389/fpubh.2023.1192517 (PMC10483823; doi:10.3389/fpubh.2023.1192517)
Supplement: Supplementary file 1 [file Table_1.docx]

# Supplementary Table 1. Years of shift work and risk of GORD

| **Years of shift work** | **HR (95% CI)** | ***P* value** |
| --- | --- | --- |
| **Model 1** |  |  |
| 1-4 y | 1.07 (0.98, 1.17) | 0.107 |
| ≥ 5 y | 1.06 (1.00, 1.11) | 0.033 |
| **Model 2** |  |  |
| 1-4 y | 1.05 (0.96, 1.14) | 0.324 |
| ≥ 5 y | 1.04 (0.99, 1.09) | 0.151 |
| **Model 2 plus all potential mediators** |  |  |
| 1-4 y | 0.95 (0.86, 1.06) | 0.357 |
| ≥ 5 y | 1.12 (1.00, 1.24) | 0.036 |

Model 1 was adjusted for age, sex, ethnicity, Townsend deprivation index, education, hours of work per week, duration of current job, walking/standing at work and heavy manual/physical work

Model 2 was further adjusted for BMI, alcohol consumption and healthy diet score
