# Supplementary material for: Shift work and risk of incident gastroesophageal reflux disease: the association and mediation
Source: Front Public Health. 2023 Aug 24;11:1192517. doi: 10.3389/fpubh.2023.1192517 (PMC10483823; doi:10.3389/fpubh.2023.1192517)
Supplement: Supplementary file 2 [file Table_2.docx]

# Supplementary Table 2. Subgroup analysis for the association of shift work and risk of GORD

|  | **HR (95% CI)** | ***P* value** | ***P* for interaction** |
| --- | --- | --- | --- |
| **Age** |  |  | 0.072 |
| <50 y | 1.07 (0.98, 1.16) | 0.129 |  |
| ≥50 y | 1.04 (0.98, 1.09) | 0.218 |  |
| **Sex** |  |  | 0.205 |
| Women | 1.07 (1.01, 1.15) | 0.035 |  |
| Men | 1.04 (0.98, 1.11) | 0.228 |  |
| **Education attainment** |  |  | 0.099 |
| Without a university degree | 1.02 (0.97, 1.08) | 0.396 |  |
| With a university degree | 1.12 (1.01, 1.24) | 0.030 |  |
| **Townsend deprivation index** |  |  | 0.550 |
| High (<–2.08) | 1.05 (0.98, 1.13) | 0.177 |  |
| Middle (≥-2.08 to <1.40) | 1.05 (0.97, 1.14) | 0.196 |  |
| Low (≥1.40) | 1.04 (0.95, 1.14) | 0.444 |  |
| **Hours of work per week** |  |  | 0.198 |
| <37 | 1.00 (0.93, 1.08) | 0.904 |  |
| ≥37 | 1.08 (1.01, 1.14) | 0.014 |  |
| **Job involves walking or standing** |  |  | 0.226 |
| Never or rarely | 1.13 (1.01, 1.27) | 0.038 |  |
| Sometimes or more | 1.03 (0.98, 1.08) | 0.265 |  |
| **Heavy manual labor** |  |  | 0.002 |
| Never/rarely | 1.14 (1.06, 1.23) | <0.001 |  |
| Sometimes or more | 0.99 (0.93, 1.05) | 0.636 |  |
| **Sleep patterns** |  |  | 0.253 |
| Poor | 0.99 (0.84, 1.17) | 0.918 |  |
| Intermediate | 1.03 (0.98, 1.09) | 0.239 |  |
| Healthy | 1.09 (0.98, 1.21) | 0.130 |  |
| **Sleep duration** |  |  | 0.985 |
| 7-8 h/d | 1.04 (0.97, 1.12) | 0.274 |  |
| <7 h/d or >8 h/d | 1.03 (0.97, 1.09) | 0.347 |  |

Models were adjusted for age, sex, ethnicity, education, Townsend deprivation index, hours of work per week, duration of current job, walking/standing at work and heavy manual/physical work.
