# Supplementary material for: Shift work and risk of incident gastroesophageal reflux disease: the association and mediation
Source: Front Public Health. 2023 Aug 24;11:1192517. doi: 10.3389/fpubh.2023.1192517 (PMC10483823; doi:10.3389/fpubh.2023.1192517)
Supplement: Supplementary file 3 [file Table_3.docx]

# Supplementary Table 3. Associations between shift work and potential mediators

| **Variable** | **β (95% CI)** | ***P* value** |
| --- | --- | --- |
| **Sleep patterns** |  |  |
| Sleep duration | -0.280 (-0.316, -0.244) | <0.001 |
| Sleep disturbance | -0.065 (-0.072, -0.051) | <0.001 |
| **Heath behaviors** |  |  |
| Current smoking | 0.064 (0.027, 0.101) | <0.001 |
| Regular exercise | -0.058 (-0.103, -0.012) | 0.013 |
| Sedentary time | 0.448 (0.407, 0.489) | <0.001 |
| **Depressive symptoms** |  |  |
| Ever felt depressed | 0.112 (0.071, 0.387) | <0.001 |
| Ever felt tense | 0.101 (0.061, 0.215) | <0.001 |
| Ever had little interest in doing things | 0.246 (0.205, 0.287) | <0.001 |
| Ever felt tired | 0.124(0.088, 0.160) | <0.001 |
| **Chronic condition** |  |  |
| Long-standing illness | 0.105 (0.064, 0.145) | <0.001 |
| Taking ≥5 medications | 0.064 (0.027, 0.100) | <0.001 |
| **Biological factors** |  |  |
| C-reactive protein | 0.074 (0.011, 0.137) | 0.021 |
| eGFR | 0.402 (0.201, 0.603) | <0.001 |
| Gamma glutamyltransferase | 0.682 (0.082, 1.283) | 0.026 |
| Grip strength | -0.548 (-0.669, -0.427) | <0.001 |
| HbA1c | 0.358 (0.257, 0.459) | <0.001 |
| Waist to hip ratio | 0.003 (0.001, 0.004) | <0.001 |

Model was further adjusted for age, sex, ethnicity, Townsend deprivation index, education level hours of work per week, duration of current job, walking/standing at work and heavy manual/physical work.

BMI: body mass index; eGFR: estimated glomerular filtration rate; GORD: gastroesophageal reflux disease; HbA1c: glycated hemoglobin
