# Supplementary material for: Shift work and risk of incident gastroesophageal reflux disease: the association and mediation
Source: Front Public Health. 2023 Aug 24;11:1192517. doi: 10.3389/fpubh.2023.1192517 (PMC10483823; doi:10.3389/fpubh.2023.1192517)
Supplement: Supplementary file 4 [file Table_4.docx]

# Supplementary Table 4. Associations between potential mediators and the risk of GORD with mediation proportions between shift work and the risk of GORD

| **Variable** | **Outcome regressed by potential mediator** | |  | **Mediation analysis** | |
| --- | --- | --- | --- | --- | --- |
|  | **HR (95% CI)** | ***P* value** |  | **% Mediated (95% CI)** | ***P* value** |
| **Sleep patterns** |  |  |  |  |  |
| Sleep duration (7–8 h/day) | 0.86 (0.83, 0.89) | <0.001 |  | 13.01 (6.72, 24.0) | <0.001 |
| Sleep disturbance per one point decrease | 0.86 (0.85, 0.88) | <0.001 |  | 9.62 (6.05, 40.00) | <0.001 |
| **Heath behaviors** |  |  |  |  |  |
| Current smoking | 1.07 (1.01, 1.13) | 0.018 |  | 4.48 (1.97, 21.22) | 0.010 |
| Regular exercise | 0.89 (0.86, 0.93) | <0.001 |  | 1.51 (0.03, 5.31) | <0.001 |
| Sedentary time per 1 hour increase per day | 1.04 (1.03, 1.05) | <0.001 |  | 15.09 (9.47, 63.23) | <0.001 |
| **Depressive symptoms** |  |  |  |  |  |
| Ever felt depressed | 1.15 (1.09, 1.21) | <0.001 |  | 11.52 (7.68, 39.80) | <0.001 |
| Ever felt tense | 1.19 (1.14, 1.25) | <0.001 |  | 9.89 (5.99, 56.77) | <0.001 |
| Ever had little interest in doing things | 1.07 (1.02, 1.13) | 0.008 |  | 11.07 (6.83, 76.22) | <0.001 |
| Ever felt tired | 1.25 (1.20, 1.30) | <0.001 |  | 9.37 (5.36, 56.81) | <0.001 |
| **Chronic condition** |  |  |  |  |  |
| Long-standing illness | 1.38 (1.33, 1.44) | <0.001 |  | 7.64 (4.25, 36.31) | <0.001 |
| Taking ≥5 medications | 1.54 (1.45, 1.64) | <0.001 |  | 4.48 (1.97, 21.16) | <0.001 |
| **Biological factors** |  |  |  |  |  |
| C-reactive protein per 1 mg/L increase | 1.01 (1.00, 1.01) | <0.001 |  | 0.32 (0.020, 1.11) | 0.041 |
| eGFR per 1 ml/min/1.73 m^2^ increase | 0.997 (0.996, 0.999) | 0.002 |  | -0.05 (-0.50, 0.01) | 0.301 |
| Gamma glutamyltransferase per 1 U/L increase | 1.0014 (1.0010, 1.0018) | <0.001 |  | 0.68 (0.0082, 4.27) | <0.001 |
| Grip strength per 1 Kg increase | 0.99 (0.98, 0.99) | <0.001 |  | 5.15 (3.06, 21.32) | <0.001 |
| HbA1c per 1 mmol/mol increase | 1.00 (0.99, 1.00) | 0.674 |  | -1.89 (-12.79, -1.11) | 0.015 |
| Waist to hip ratio per 1 unit increase | 10.92 (8.38, 14.23) | <0.001 |  | 3.52 (1.88, 15.01) | <0.001 |

Model was adjusted for shift work status, age, sex, ethnicity, Townsend deprivation index, education level, hours of work per week, duration of current job, walking/standing at work and heavy manual/physical work.

BMI: body mass index; eGFR: estimated glomerular filtration rate; GORD: gastroesophageal reflux disease; HbA1c: glycated hemoglobin
