# Supplementary material for: Shift work and risk of incident gastroesophageal reflux disease: the association and mediation
Source: Front Public Health. 2023 Aug 24;11:1192517. doi: 10.3389/fpubh.2023.1192517 (PMC10483823; doi:10.3389/fpubh.2023.1192517)
Supplement: Supplementary file 5 [file Data_Sheet_1.docx]

# Supplementary Material 1. Details of the covariates

## Sleep patterns

Circadian was defined as the chronotype preference, assessed by the question: ‘Do you consider yourself to be (i) definitely a “morning” person, (ii) more a “morning” than “evening” person, (iii) ‘more an “evening” than “morning” person’, or (iv) definitely an “evening” person’. In the present study, the option “definitely a “morning” person” was combined into “more a “morning” than “evening” person”, resulting in a group “early chronotype”; similarly, the other options were combined into a new group “late chronotype”. Sleep duration was categorized as normal (7–8 h/day), or abnormal (>9 h/day or <7 h/day) as for previous research (1, 2, 3, 4). Insomnia symptoms were determined by the question: ‘Do you have trouble falling asleep at night or do you wake up in the middle of the night?’ with options of (i) never/rarely, (ii) sometimes, or (iii) usually. Snoring was collected by asking ‘Does your partner or a close relative or friend complain about your snoring?’ with responses of (i) yes or (ii) no. Subjective daytime sleepiness was collected by the question ‘How likely are you to doze off or fall asleep during the daytime when you don’t mean to? (e.g. when working, reading or driving)’ with responses of (i) never/rarely, (ii) sometimes, (iii) often, or (iv) all of the time.

As suggested by previous studies, chronotype reflects one’s circadian, while other sleep behaviors represent the sleep quality (5, 6). The number of sleep disturbances included five circadian and sleep characteristics: late chronotype, inadequate sleep duration (7-8 hours/day), usually insomnia, snoring, and frequent daytime sleepiness.

## Healthy diet score

The healthy diet score was calculated using the medians of several dietary components as follows: red meat intake less than three times each week; vegetable intake at least four tablespoons each day; fruit intake of at least three pieces each day; fish intake of at least four times each week; cereal intake of at least five bowls each week; and urinary sodium concentration (measured in stored urine samples using ionselective electrode method) less than 68.3 mmol/l. Each favourable diet factor was assigned a score of 1 point, resulting in a total diet score ranging from 0 to 6 (7). As such, a higher score may represent a healthier diet pattern.

## Alcohol consumption

Alcohol consumption was estimated by 18 questions regarding intakes of beer, wine (red and white) and spirits. Specifically, participants were asked how often they drank alcohol with answers being: “daily or almost daily”, “three or four times a week”, “once or twice a week”, “one to three times a month”, “special occasions only”, “never”, “prefer not to answer”. Additionally, they were also asked separately about weekly and monthly consumption of pints of beer, glasses of red wine, glasses of white wine/champagne, glasses of fortified wine, measures of spirits/liqueurs and glasses of other alcohol. We assumed a pint of beer contained 20 g of alcohol, and all other drinks contained 10 g, and summed their total weekly and monthly consumption of alcohol according to a published study of the UK Biobank (8).

## Regular physical activity

Physical activity was defined as regular if participants reported ≥150 min/wk moderate or ≥75 min/wk vigorous or ≥150 min/wk mixed activity (9), or ≥5 days/week of moderate physical activity 10+ minutes or ≥3 days/week of vigorous physical activity 10+ minutes.

## Sedentary time

Sedentary time (hours) was calculated by the summing the scores of three questions about the hours per day participants spent (1) driving, (2) using a computer, and (3) watching television.

# Reference

1. Li X, Zhou T, Ma H, Huang T, Gao X, Manson JE, et al. Healthy sleep patterns and risk of incident arrhythmias. Journal of the American College of Cardiology. 2021;78(12):1197-207.

2. Itani O, Jike M, Watanabe N, Kaneita Y. Short sleep duration and health outcomes: a systematic review, meta-analysis, and meta-regression. Sleep medicine. 2017;32:246-56.

3. Huang B-H, Duncan MJ, Cistulli PA, Nassar N, Hamer M, Stamatakis E. Sleep and physical activity in relation to all-cause, cardiovascular disease and cancer mortality risk. British Journal of Sports Medicine. 2021.

4. Fan M, Sun D, Zhou T, Heianza Y, Lv J, Li L, et al. Sleep patterns, genetic susceptibility, and incident cardiovascular disease: a prospective study of 385 292 UK biobank participants. European heart journal. 2020;41(11):1182-9.

5. Li Z-H, Zhang P-D, Chen Q, Gao X, Chung VC, Shen D, et al. Association of sleep and circadian patterns and genetic risk with incident type 2 diabetes: a large prospective population-based cohort study. European journal of endocrinology. 2021;185(5):765-74.

6. Tan X, Ciuculete DM, Schiöth HB, Benedict C. Associations between chronotype, MTNR1B genotype and risk of type 2 diabetes in UK Biobank. Journal of internal medicine. 2020;287(2):189-96.

7. Li FR, He Y, Yang HL, Liu HM, Zhou R, Chen GC, et al. Isolated systolic and diastolic hypertension by the 2017 American College of Cardiology/American Heart Association guidelines and risk of cardiovascular disease: a large prospective cohort study. J Hypertens. 2021;39(8):1594-601.

8. Bradbury KE, Murphy N, Key TJ. Diet and colorectal cancer in UK Biobank: a prospective study. International journal of epidemiology. 2020;49(1):246-58.

9. Said MA, Verweij N, van der Harst P. Associations of combined genetic and lifestyle risks with incident cardiovascular disease and diabetes in the UK Biobank Study. JAMA cardiology. 2018;3(8):693-702.
